# Fruit and vegetable consumption in the former Soviet Union: the role of individual- and community-level factors

**DOI:** 10.1017/S1368980015000105

**Published:** 2015-02-17

**Authors:** Yevgeniy Goryakin, Lorenzo Rocco, Marc Suhrcke, Bayard Roberts, Martin McKee

**Affiliations:** 1 Norwich Medical School, University of East Anglia, Norwich NR4 7TJ, UK; 2 UKCRC Centre for Diet and Activity Research (CEDAR), Cambridge, UK; 3 Department of Economics, University of Padua, Padua, Italy; 4 Centre for Health Economics, University of York, York, UK; 5 European Centre on Health of Societies in Transition, Department of Health Services Research and Policy, London School of Hygiene and Tropical Medicine, London, UK

**Keywords:** Nutrition, Fruit and vegetable consumption, Socio-economic determinants

## Abstract

**Objective:**

To explain patterns of fruit and vegetable consumption in nine former Soviet Union countries by exploring the influence of a range of individual- and community-level determinants.

**Design:**

Cross-sectional nationally representative surveys and area profiles were undertaken in 2010 in nine countries of the former Soviet Union as part of the Health in Times of Transition (HITT) study. Individual- and area-level determinants were analysed, taking into account potential confounding at the individual and area level.

**Setting:**

Armenia, Azerbaijan, Belarus, Georgia, Kazakhstan, Kyrgyzstan, Moldova, Russia and Ukraine.

**Subjects:**

Adult survey respondents (*n* 17 998) aged 18–95 years.

**Results:**

Being male, increasing age, lack of education and lack of financial resources were associated with lower probability of consuming adequate amounts of fruit or vegetables. Daily fruit or vegetable consumption was positively correlated with the number of shops selling fruit and vegetables (for women) and with the number of convenience stores (for men). Billboard advertising of snacks and sweet drinks was negatively related to daily fruit or vegetable consumption, although the reverse was true for billboards advertising soft drinks. Men living near a fast-food outlet had a lower probability of fruit or vegetable consumption, while the opposite was true for the number of local food restaurants.

**Conclusions:**

Overall fruit and vegetable consumption in the former Soviet Union is inadequate, particularly among lower socio-economic groups. Both individual- and community-level factors play a role in explaining inadequate nutrition and thus provide potential entry points for policy interventions, while the nuanced influence of community factors informs the agenda for future research.

The publication of the 2010 Global Burden of Disease Study reinforced the importance of adequate fruit and vegetable consumption^(^ ^1^ ^)^, primarily via its impact on cardiovascular health^(^ ^2^ ^)^ and some cancers^(^ ^3^ ^,^ ^4^ ^)^. The WHO and the FAO recommend a minimum fruit and vegetable consumption of at least 400 g/d for adults^(^ ^5^ ^)^.

The volume of research on determinants of fruit and vegetable consumption in high-income countries^(^ ^6^ ^–^ ^8^ ^)^ is not matched by its scarcity in the countries of the former Soviet Union (FSU), even though global agricultural trade data suggest that consumption there is especially low^(^ ^4^ ^)^. One survey found fruit and vegetable consumption to be inadequate (defined as eating less than 400 g/d, or five servings of 80 g/d) among 80 % of people in Russia, 92 % in Kazakhstan and 55 % in Ukraine^(^ ^9^ ^)^. Another study found that 93 % of men living in Russian Karelia consumed inadequate vitamin C, compared with only 2 % in neighbouring Finnish Karelia^(^ ^10^ ^)^, subsequently linked to low fruit consumption^(^ ^11^ ^)^.

Although studies of environmental determinants of dietary consumption have increased globally, the existing evidence remains insufficient to draw robust conclusions^(^ ^12^ ^)^ and what does exist is limited in scope. Brug^(^ ^13^ ^)^ has contrasted the relative lack of evidence on macro-level environmental determinants of nutrition with that on micro-level determinants, with most research worldwide having focused on biological, psychological, behavioural and social factors acting at the individual level^(^ ^13^ ^)^. However, there is growing interest in the role of environmental determinants^(^ ^14^ ^,^ ^15^ ^)^ as the explanatory power of individual factors alone has proved limited^(^ ^16^ ^)^.

By assessing both individual- (e.g. age, gender, marital and socio-economic status) and community-level (e.g. advertising for high-energy food and drinks, availability of shops selling fruit and vegetables, ease of access to fast-food outlets) drivers of fruit and vegetable consumption in nine FSU countries, the present study contributes to the global body of research on determinants of diet and obesity. Our aims are: (i) to present new estimates of the prevalence of fruit and vegetable consumption in nine FSU countries; and (ii) to identify relevant individual- and community-level determinants.

## Methods

### Study design

Data are from household surveys in nine countries of the FSU as part of the Health in Times of Transition (HITT) study^(^ ^17^ ^)^. This used the same standardized questionnaire in each country to capture a range of health outcomes, health behaviours and demographic, socio-economic and environmental characteristics. Surveys were nationally representative and conducted among adult respondents (aged ≥18 years) in Armenia, Azerbaijan, Belarus, Georgia, Kazakhstan, Kyrgyzstan, Moldova, Russia and Ukraine.

Multistage random sampling with stratification by region and rural/urban settlement type was applied. Sample size for the urban and rural population was determined proportionally to these populations in each study country. Primary sampling units were selected randomly using the probability-proportional-to-size technique from routine data. Within each primary sampling unit (about 100–200 per country, except Russia and Ukraine with 329 and 435 primary sampling units, respectively) households were selected by random route procedures. Within each selected household one person was chosen (based on nearest birthday). If after three visits (on different days and times) there was no one at home, the next household on the route was selected.

The surveys were conducted between March and May 2010, except in Kyrgyzstan where there was a delay until March to May 2011 due to political violence. Face-to-face interviews were conducted by trained fieldworkers in respondents’ homes. Response rates varied from 47·3 % in Kazakhstan to 82·9 % in Georgia. Each country had 1800 respondents, except Russia (*n* 3000) and Ukraine (*n* 2200) to reflect their larger and more regionally diverse populations, and Georgia (*n* 2200) where a booster survey of 400 additional interviews was undertaken in November 2010 to ensure a more representative sample. The final sample used in the individual-level regression analysis was slightly smaller due to a small number of missing observations. The sample that was used in the community-level analysis was considerably smaller due to the fact that only a sub-sample of communities was selected for data collection. However, since the communities were randomly drawn from the larger number of sampling units used in the main HITT household survey, there is unlikely to be any bias introduced by this drop in the sample size (as the individual observations are also missing at random).

The draft questionnaire was forward- and backward-translated into each of the languages in which it was administered, and then piloted with approximately fifteen people in each country. Except in Russia and Belarus (where all interviews were conducted in Russian), respondents were given the choice of answering in Russian or a national language.

The research was approved by the ethics committee of the London School of Hygiene and Tropical Medicine and was conducted in accordance with the ethical standards laid down in the 1964 Declaration of Helsinki. All persons gave informed consent. Quality control procedures included re-interviews to assess the work of both the interviewers and the interviewers’ supervisors.

### Variables

Our main dependent variable was daily or almost daily consumption of fruit or vegetables (excluding potatoes). This ranges from 1, referring to daily or almost daily consumption, to 4, referring to consuming fruit or vegetables less than once weekly (with information only on the frequency of consumption being available, and not on the quantity).

Our independent variables included indicators for people who believe that good diet is unimportant, age, being female, having primary or secondary levels of education as the highest attainment, reporting good economic status, number of people in the household, being married, asset classes and living in the rural area. More details are given in the online supplementary material, Annex 1 and Annex 2.

Additional community-level variables were recorded in a sub-sample of 333 primary sampling units randomly selected from those in the main household surveys. The community-level variables were measured using a standardized Community Observation Form, based upon the validated Prospective Urban and Rural Epidemiology Study’s Environmental Profile of a Community’s Health (EPOCH) instrument^(^ ^18^ ^)^. Two trained data collectors per community systematically recorded aspects of the environment relating to general social/economic situation (e.g. community-specific architecture such as conditions of homes and roads), nutrition and physical activity (e.g. walkability and food environment) and tobacco and alcohol (e.g. availability and advertising). Thirty community profiles were conducted in each country, except Russia (seventy-three profiles) and Ukraine (fifty profiles) to reflect their larger and more regionally diverse populations. Additional information on the community profile instrument is available elsewhere^(^ ^19^ ^)^.

We used data on the number of outdoor advertisements (e.g. billboards, adverts on shop windows, bus shelters and other easily accessible locations) for fast foods, snacks, fizzy carbonated drinks, as well as for sweet drinks (including juices). These were collected independently of the interviews.

Community-level data also included the number of shops/other outlets selling sweets, biscuits and crisps, as well as fruit and vegetables, with kiosks being included in this category. Incidentally, in the FSU kiosks rarely appear to sell fruit and vegetables and instead appear primarily as outlets for alcohol and tobacco^(^ ^20^ ^)^. Finally, information was obtained on whether people lived within an ‘easy walk’ to fast-food outlets within a community, as well as on the number of restaurants and cafés in the community selling local food. Cafeterias were counted separately from fast-food restaurants.

All variables used in the analysis were based on closed-ended questions, except the information on the number of advertisements/outlets, collected by two observers per community.

### Statistical analysis

We examined the statistical association between fruit and vegetable consumption and potential individual- and community-level determinants. Analyses were conducted using the statistical software package Stata 11. We began the analysis with ordinary least squares (OLS) regression, where the outcome variable was daily or almost daily consumption of either fruit or vegetables, and performed the regression of the outcome variable *v*. individual-level covariates and country dummies. We then controlled for potential confounding with community fixed effects (CFE).

Next, as our interest shifted to estimating the association between several community-level variables and fruit or vegetable consumption, we performed the regression of this outcome *v*. the set of community-level covariates of interest, as described below. To reduce the potential for confounding, we controlled for several potentially relevant community-level variables, as well as for regional fixed effects.

Finally, to take advantage of the ordered nature of the underlying variables used to define consumption, ordered probit results were estimated, separately for fruit and vegetable consumption. As the underlying outcome variable in this specification ranges from 1 to 4, with the value of 1 referring to eating fruit or vegetables less than once weekly and 4 to eating daily or almost daily, positive ordered probit estimates indicate a greater probability of eating fruit or vegetables and, conversely, negative estimates are associated with factors that reduce the likelihood of consuming fruit and vegetables. Although, in principle, one may apply multinomial logit or probit models to estimate effect of covariates on these outcomes, ordered probit specification takes advantage of the natural ordering of the data, also allowing a more parsimonious presentation of results^(^ ^21^ ^)^.

The initial specification was as follows:(1)
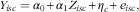
where *Y* is the dummy variable for individual *i* living in community *s* located in country *c*, with the value of 1 assigned to individuals reporting daily or almost daily consumption of either fruit or vegetables. In equation (1), the main interest is in the parameters contained in a vector *α*_1_ obtained from regression of the outcome variable *Y*_*isc*_ *v*. the vector of individual-level determinants *Z*_*isc*_ and country effects *η*_c_, as described in the ‘Variables’ section.

To control for additional area-level confounders that affect both the covariates of interest included in *Z*_*isc*_ and the outcome variable, a richer specification was considered that replaced country with community fixed effects. For example, community-level infrastructure and employment opportunities may be a determinant of both fruit and vegetable consumption, as well as of reporting good health and of good economic status. Also, to control for any correlation of the error term *e*_*isc*_ among individuals belonging to the same community, we clustered standard errors on the community level.

Next, the association of community-level determinants with the same outcome of interest was estimated according to the following specification:(2)
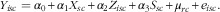



The parameters contained in vector *α*_1_ are associated with a vector of community determinants (also used as simultaneous controls), 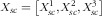, that included three sets of community determinants:

1.

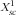
 included variables measuring exposure to different types of advertising for high-energy foods and drinks. As an *ad hoc* hypothesis, it is expected that greater exposure to these advertisements will negatively affect the probability of daily fruit and vegetable consumption.
2. With 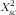, the focus was on availability of healthy and unhealthy foods in stores. *A priori*, one expects fruit and vegetable outlets to increase availability of those products, as well as to positively affect preferences for their greater consumption, while the reverse will be true for stores selling sweets and crisps.
3. Finally, in 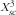, the focus was on outside eating establishments, such as ease of access to fast-food outlets and general service restaurants. Our *a priori* expectation is that easier access to fast-food outlets will be associated with worse dietary attitudes and lower fruit and vegetable consumption; at the same time it is not clear what association to expect between our outcomes of interest and the number of local restaurants.

The main problem with estimating equation (2) is potential area-level confounding. For example, some previous research found fruit and vegetable consumption to be positively correlated with neighbourhood average income^(^ ^7^ ^)^, but wealthier neighbourhoods may also have better access to supermarkets and a wider variety of foods^(^ ^22^ ^)^. Taken together, this evidence suggests that area-level socio-economic status may drive the observed association between dietary outcomes of interest and environmental determinants, by affecting both simultaneously.

To control for potential area-level socio-economic confounders, we included a vector of neighbourhood control variables *S*_*sc*_ in equation (2), such as dummy variables for: living in the capital city; living in communities where garbage is collected by authorities from all homes; living in communities where all homes have a cold water supply; living in communities where all homes have a central heating system; and living in communities where there are no derelict homes present. In addition, regional fixed effects *μ*_*rc*_ were included in equation (2) which account for potential confounders that vary at that geographic level. Differently from equation (1), community fixed effects cannot be included as they will be perfectly collinear with the vector *X*_*sc*_.

Finally, a vector of individual-level determinants *Z*_*isc*_ accounted for the remaining variation at the individual level.

## Results

### Descriptive statistics

Table 1 presents the main descriptive statistics. In all countries, the proportion of people consuming fruit or vegetables daily or several times weekly exceeded the proportion of people eating them once weekly or less than once weekly. This was also confirmed in formal tests (results available upon request), as the *P* value is in all cases less than 0·001.

**Table 1:** Selected descriptive statistics, by country

	Armenia	Azerbaijan	Belarus	Georgia	Kazakhstan	Kyrgyzstan	Moldova	Russia	Ukraine
Response rate (%)	60·1	56·8	48·1	82·9	47·3	78·4	74·8	59·2	60·1
Fruit or vegetable consumption									
Men and women									
Daily (%)	49·8	55·5	32·2	32·6	47·4	36·9	30·4	42·7	48·9
Several times weekly (%)	36·2	29·5	42·9	41·7	30·8	33·4	34·2	42·3	34·3
Once weekly (%)	12·1	12·8	15·4	15·9	13·7	16·6	16·8	12·5	11·2
Less than once weekly (%)	1·9	2·2	9·6	9·8	8·1	13·0	18·6	2·6	5·6
Women only									
Daily (%)	50·4	55·8	34·8	34·0	49·5	37·6	30·7	45·1	49·7
Several times weekly (%)	35·1	30·0	43·1	40·4	29·3	31·6	34·2	42·2	33·7
Once weekly (%)	12·3	11·7	13·7	15·2	13·4	17·4	16·9	10·6	10·7
Less than once weekly (%)	2·2	2·4	8·4	10·4	7·9	13·3	18·2	2·1	6·0
Men only									
Daily (%)	49·1	55·0	28·8	30·1	45·0	36·2	30·0	39·1	47·8
Several times weekly (%)	37·5	29·0	42·6	44·1	32·4	35·4	34·2	42·3	35·2
Once weekly (%)	11·8	14·2	17·4	17·0	14·2	15·7	16·7	15·3	11·8
Less than once weekly (%)	1·6	1·8	11·1	8·9	8·4	12·6	19·1	3·3	5·2
Community-level determinants									
No. of fast-food adverts	3·0	3·5	0·0	0·5	0·1	0·5	3·5	0·6	1·3
No. of snack adverts	2·4	4·5	1·1	0·5	0·5	0·3	3·0	2·0	1·8
No. of soft drink adverts	2·4	2·3	2·3	2·0	0·7	1·1	3·7	2·0	4·5
No. of sweet drink/juice adverts	4·0	2·0	3·3	2·5	1·8	2·3	9·1	3·3	4·5
No. of shops selling crisps and sweets	6·5	6·5	8·8	6·9	6·7	6·7	9·7	7·9	8·2
No. of shops selling fruit and vegetables	4·9	5·0	8·2	3·4	4·8	4·1	6·4	6·1	5·0
No. of local restaurants	3·4	2·4	2·7	2·2	2·6	0·8	4·9	1·9	2·8

Source: Health in Times of Transition (HITT) data set, 2010. In all columns, mean values are presented. Summary of individual-level data represents average proportion of people eating fruit or vegetables daily, several times weekly, once weekly and less than once weekly. Community-level data represent mean values per community, weighted by community size. Each community represents a separate primary sampling unit, equivalent to a ‘rayon’ or a small administrative region.

However, in only one country (Azerbaijan) did more than half of the surveyed population eat fruit or vegetables daily/almost daily; in four other countries this proportion was about one-third. Table 1 shows that the gender difference was relatively small except in Belarus (34·8 % for women *v*. 28·8 % for men) and Russia (45·1 % for women *v*. 39·1 % for men). Weighted average values for community variables used in the analysis are also presented in Table 1 (the weights are numbers of respondents living in respective communities).

### Individual determinants

Our main ordinary least squares regression results are presented in Table 2, for the whole sample and separately for men and women (columns 1–3). Each year of age reduced the probability of daily/almost daily fruit or vegetable consumption by about 0·1 %.

**Table 2:** Individual-level determinants of daily/almost daily fruit or vegetable consumption in nine countries of the former Soviet Union

	Column 1		Column 2		Column 3		Column 4		Column 5		Column 6	
	All		Women		Men		All		Women		Men	
	Coefficient†	se	Coefficient†	se	Coefficient†	se	Coefficient‡	se	Coefficient‡	se	Coefficient‡	se
Good diet not important	0·032	0·045	0·025	0·063	0·041	0·057	−0·024	0·038	0·043	0·054	−0·077	0·055
Age	−0·001***	0·000	−0·001***	0·000	0·000	0·000	−0·001***	0·000	−0·002***	0·000	−0·001**	0·000
Female	0·041***	0·008	–	–	–	–	0·039***	0·007	–	–	–	–
Primary education	−0·072***	0·015	−0·073***	0·020	−0·066***	0·020	−0·063***	0·013	−0·078***	0·018	−0·037*	0·021
Secondary education	−0·054***	0·010	−0·053***	0·013	−0·050***	0·014	−0·035***	0·009	−0·051***	0·012	−0·017	0·014
Good economic situation	0·081***	0·012	0·065***	0·015	0·098***	0·016	0·079***	0·010	0·073***	0·014	0·080***	0·015
Household size	0·000	0·003	−0·002	0·003	0·002	0·004	−0·002	0·002	−0·005*	0·003	0·001	0·004
Married	0·023***	0·008	0·015	0·011	0·026*	0·013	0·031***	0·007	0·014	0·010	0·046***	0·013
Village	−0·018	0·016	−0·006	0·018	−0·033*	0·019	–	–	–	–	–	–
Capital	0·056***	0·020	0·053**	0·023	0·062**	0·024	–	–	–	–	–	–
Asset2	0·020	0·012	0·019	0·015	0·019	0·017	0·025**	0·011	0·032**	0·015	0·013	0·017
Asset3	0·056***	0·013	0·058***	0·017	0·050***	0·019	0·053***	0·011	0·063***	0·016	0·032*	0·018
Asset4	0·114***	0·014	0·135***	0·018	0·091***	0·019	0·106***	0·011	0·132***	0·016	0·070***	0·018
No. of observations	17 305		9778		7527		17305		9778		7527			

Source: Health in Times of Transition (HITT) data set, 2010. Cluster-robust standard errors are presented. All specifications also include country dummies. *Significant at 10 % level; **significant at 5 % level; ***significant at 1 % level.

† Ordinary least squares (OLS) model.

‡ Community fixed effects (CFE) model.

It has already been shown in Table 1 that women tended to consume more fruit and vegetables in all countries, a finding confirmed in the multivariate analysis, with women having about 4 % greater probability of eating fruit or vegetables daily compared with men. Education was positively correlated with daily consumption of fruit or vegetables, with people with tertiary education being 5·4 % more likely to eat them daily, compared with those with secondary education only. Reporting a good financial situation (even controlling for wealth) was associated with about 8 % greater probability of eating fruit or vegetables daily. Similarly, people whose combined wealth placed them in the top 25 % of the asset score in their countries were about 11 % more likely to report daily fruit or vegetable consumption, compared with the bottom 25 %. Living in the capital was associated with about 5·6 % higher probability of reporting daily fruit or vegetable consumption. Being married was significantly positively related to the probability of reporting daily fruit or vegetable consumption. Finally, the perception that diet is not important to good health was unrelated to fruit or vegetable consumption.

Being older reduced the probability of eating fruit or vegetables daily by women, but not by men (Table 2, columns 2–3). On the other hand, the role of economic situation appeared stronger among men than among women. Being married was related to greater likelihood of eating fruit or vegetables daily, but only by men. Other associations were similar for men and women.

As a robustness check, community fixed effects were included in equation (1), as their inclusion should control for local heterogeneity more precisely. The estimates, reported in Table 2, columns 4–6, show that there was little difference compared with the baseline estimates (columns 1–3), although parameters tended to be somewhat smaller (but still significant).

### Community determinants

Table 3 presents the main community-level parameters. The number of snack and sweet drink advertisements in the community was significantly negatively related to the probability of eating fruit or vegetables daily (Table 3, column 1). An additional advertisement for snacks reduced this outcome by about 3 %, while another advertisement for sweet drinks reduced it by about 1·6 %. This association was significant (and of much larger in size) only for women, compared with men (compare columns 2 and 3). Living within an easy walk to a fast-food outlet was associated with a very large (16·1 %) reduction in the probability of eating fruit or vegetables daily for men (although the association was insignificant for women). Similarly, more shops selling fruit and vegetables was positively correlated with the probability of eating fruit or vegetables daily, although only significantly so for women. Furthermore, more restaurants in the community was positively correlated with daily consumption of either fruit or vegetables (although only significantly so for women). Finally, a greater number of soft drink adverts, as well as a greater number of shops selling crisps and sweets, was positively related to daily fruit or vegetable consumption (in the former case, the effect was significant among women; in the latter, among men).

**Table 3:** Community-level determinants of daily/almost daily fruit or vegetable consumption in nine countries of the former Soviet Union

	All		Women		Men	
	Coefficient†	se	Coefficient†	se	Coefficient†	se
Food adverts	0·020	−0·018	0·028	−0·020	0·013	−0·025
Snack adverts	−0·031***	−0·011	−0·040***	−0·011	−0·019	−0·015
Soft drink adverts	0·023*	−0·012	0·032***	−0·011	0·027	−0·017
Sweet drink/juice adverts	−0·016*	−0·008	−0·025**	−0·011	−0·015	−0·009
Shops selling crisps and sweets	0·005	−0·008	−0·011	−0·009	0·022**	−0·009
Shops selling fruit and vegetables	0·009	−0·010	0·023**	−0·012	−0·004	−0·012
No. of local food restaurants	0·015	−0·009	0·029**	−0·012	−0·005	−0·010
Easy walk to fast-food outlet	−0·069	−0·046	−0·012	−0·064	−0·161**	−0·062
No. of observations	1680		946		734	

Source: Health in Times of Transition (HITT) data set, 2010. Cluster-robust standard errors are presented. *Significant at 10 % level; **significant at 5 % level; ***significant at 1 % level.

† Ordinary least squares (OLS) parameter estimates are presented. All specifications include the same control variables as in Table 2. In addition, all specifications include the following community control variables: dummy indicators for living in communities where no homes have garbage collected by authorities from all homes; for living in communities where no homes have a cold water supply; for living in communities where no homes have a central heating system; and for living in communities where there are no derelict homes present. Finally, all specifications include regional fixed effects.

### Additional checks

Table 4 presents the ordered probit results. In columns 1 and 2, the focus is on individual-level determinants; while in columns 3 and 4 community variables are added. Age was negatively related to frequent fruit or vegetable consumption. Conversely, women were more likely to eat fruit or vegetables more frequently. These parameters were also positive for education, good economic status, being married, greater wealth and negative for living in a village. Finally, all community-level parameters were now insignificant (but recall that the outcome is defined differently from that in Table 3 and that results are now presented separately for fruit and vegetable consumption).

**Table 4:** Ordered probit results for fruit or vegetable consumption† in nine countries of the former Soviet Union, without and with community determinants

	Column 1		Column 2		Column 3		Column 4	
	Vegetables		Fruit		Vegetables‡		Fruit‡	
	Coefficient	se	Coefficient	se	Coefficient	se	Coefficient	se
Good diet not important	−0·071	0·103	0·032	0·106	0·265	0·363	0·072	0·293
Age	−0·002***	0·001	−0·004***	0·001	−0·002	0·002	−0·004**	0·002
Female	0·073***	0·017	0·111***	0·017	−0·003	0·053	0·091	0·060
Primary education	−0·226***	0·035	−0·226***	0·036	−0·079	0·099	−0·166	0·112
Secondary education	−0·147***	0·022	−0·185***	0·022	−0·155**	0·065	−0·180***	0·069
Good economic situation	0·186***	0·029	0·298***	0·028	0·219***	0·078	0·370***	0·070
Household size	0·002	0·006	0·005	0·007	0·004	0·023	0·010	0·017
Married	0·060***	0·019	0·066***	0·019	0·057	0·062	0·043	0·060
Village	−0·077**	0·038	−0·209***	0·035	0·066	0·120	0·022	0·115
Capital	0·074	0·049	0·108**	0·047	0·318**	0·161	0·250*	0·144
Asset2	0·084***	0·028	0·173***	0·029	0·099	0·084	0·216**	0·085
Asset3	0·170***	0·03	0·279***	0·031	0·149	0·092	0·202**	0·093
Asset4	0·334***	0·032	0·390***	0·033	0·347***	0·094	0·418***	0·102
No. of food adverts	–	–	–	–	0·049	0·039	−0·030	0·032
No. of snack adverts	–	–	–	–	−0·037	0·026	0·007	0·023
No. of soft drink adverts	–	–	–	–	0·033	0·028	0·007	0·021
No. of juice adverts	–	–	–	–	−0·007	0·017	0·021	0·014
No. of shops selling sweets and crisps	–	–	–	–	−0·003	0·016	−0·012	0·011
No. of shops selling fruit and vegetables	–	–	–	–	0·009	0·02	0·022	0·014
Number of local food restaurants	–	–	–	–	0·009	0·016	−0·017	0·013
Easy walk to fast food outlet	–	–	–	–	−0·097	0·103	−0·011	0·099
Observations	17 395		17 372		1692		1685	

Source: Health in Times of Transition HITT data set, 2010. Original parameters are presented. Cluster-robust standard errors are presented. *Significant at 10 % level; **significant at 5 % level; ***significant at 1 % level.

† The outcome variable range is from 1=less than once weekly to 4=daily/almost daily.

‡ Specifications include the following community control variables: dummy variables for living in communities where garbage is collected by authorities from all homes; for living in communities where all homes have a cold water supply; for living in communities where all homes have a central heating system; and for living in communities where there are no derelict homes present.

## Discussion

### Prevalence of fruit and vegetable consumption

Overall, fruit or vegetable consumption in the FSU appears inadequate, consistent with other evidence from this region^(^ ^9^ ^,^ ^23^ ^)^. However, it should be noted that existing studies in the FSU region only cover some countries and do not examine determinants of dietary patterns. One exception is a recent study^(^ ^17^ ^)^ which found that fruit and vegetable consumption in eight former Soviet countries has worsened in the past decade, especially among the poor and those in rural areas. However, it is much more descriptive than ours. While we consider community-level determinants in addition to individual ones, that study did not take advantage of the community-level data set. It also focused mainly on the determinants of inadequate fruit and vegetable consumption (i.e. fruit once weekly or less often, or vegetables once weekly or less often), while we consider determinants of good (i.e. daily/almost daily fruit or vegetable consumption). Another (less important) difference is that the previous study considered prevalence and determinants of fruit and vegetable consumption separately by fruit and vegetables, while we aggregated consumption. Our approach is more relevant in our view, because international guidelines prescribe combined fruit and vegetable consumption of 400 g/d, rather than separately for fruit or vegetables.

Some findings are unexpected: despite its large agricultural sector and warm climate, Moldova has the smallest proportion of people reporting fruit or vegetable consumption more than once weekly. This may be because it is one of the poorest countries in the FSU, with a rapidly growing share of its agricultural output now being exported^(^ ^24^ ^)^. Interestingly, only 0·1 % of respondents living in Moldova agreed with the statement that good diet is not important for health (see online supplementary material, Annex 3). At the same time, in Russia, where a relatively large proportion of people reported daily fruit or vegetable consumption, 1·85 % (the highest number) agreed with this statement. This gap between the perceived importance of good diet and actual fruit or vegetable consumption merits further study, although research elsewhere has found a similar disconnect between knowledge and practice^(^ ^25^ ^)^. It should also be noted that these proportions are estimated for a very small number of respondents; fifty-four out of 2922 in the case of Russia, for example.

### Socio-economic and demographic determinants

Our findings are consistent with other evidence on the social patterning of fruit and vegetable consumption (i.e. according to socio-economic status)^(^ ^7^ ^,^ ^26^ ^–^ ^29^ ^)^. Thus, variables such as education, household economic situation and household size, as well as wealth, are all independently associated with daily fruit or vegetable consumption in the FSU.

Similarly, the lower probability of daily fruit or vegetable consumption with increasing age is consistent with some previous studies^(^ ^30^ ^)^ but not all^(^ ^28^ ^)^. While older people may have less disposable income to spend on nutritious food, it is also likely that age has an independent effect, as a range of socio-economic variables are controlled for in all regression results reported in Table 1. One potential explanation is that older people living in the FSU may prefer to eat more traditional diets, which in many countries in that region are based on meat and carbohydrate-rich foods such as potatoes and grains.

Like us, some previous studies also found that women, and those who are married, are more likely to eat enough fruit and vegetables^(^ ^7^ ^,^ ^28^ ^)^, including in Russia and several Central and Eastern European countries^(^ ^31^ ^,^ ^32^ ^)^. This is in line with findings that in Russia, for example, women are much less likely to engage in dangerous health behaviours such as smoking and excessive alcohol consumption^(^ ^33^ ^)^, which suggests that women living in the FSU may be more health-conscious then men. Since in that region, women traditionally spend more time cooking than their husbands, this may also explain why married people are more likely to eat healthily.

Few studies have examined how fruit and vegetable consumption varies among those living in rural and urban areas. One study from the USA found people in rural areas more likely to consume fruit and vegetables^(^ ^34^ ^)^; in contrast, a European study found living in rural areas to be associated with lower consumption^(^ ^7^ ^)^. There is no significant association in the ordinary least squares models reported in Table 2, but living in rural areas is negatively related to fruit or vegetable consumption in the ordered probit regression model (Table 4). This finding may look somewhat counterintuitive but again one possible explanation is the preference for the traditional diet rich in grains, potato and meat (recall that potatoes are excluded from the definition of vegetable consumption).

### Food stores and supermarkets

Theoretically, greater availability of food stores and supermarkets may increase access to fruits and vegetables, thus contributing to increased consumption, for reasons such as lower travel and time costs of obtaining such foods; stimulation of consumption by visual cues; and the effect of exposure on food preference^(^ ^35^ ^)^. However, better access to supermarkets and food stores may also provide greater exposure to unhealthy foods and therefore, *a priori*, the overall effect is far from clear.

The available evidence does not clearly support the assertion that better access to food stores improves fruit and vegetable consumption^(^ ^36^ ^)^. However, most of the existing evidence is derived from cross-sectional studies^(^ ^37^ ^)^ conducted in high-income countries, so their findings may not be transferable to poorer countries in the FSU. Adding to the complexity, several studies found consistent positive associations between healthy dietary patterns and supermarket access in the USA^(^ ^38^ ^)^, but not in Europe^(^ ^39^ ^)^. One potential explanation is the greater locational segregation in the USA^(^ ^38^ ^)^, with supermarkets distributed more evenly among poor and wealthy districts in Europe, or because of better access to retail food outlets in Europe due to better public transport.

Although our data do not capture the number of supermarkets in the neighbouring area, they show that access to shops selling fruit and vegetables is positively and significantly correlated with daily fruit or vegetable consumption for women. One potential explanation is that it is not really the proximity of additional stores selling fruit and vegetables that influences fruit and vegetable consumption, but rather the fact that they are situated in wealthier areas, where people may be better educated about the importance of nutritious food and have higher incomes to purchase them^(^ ^22^ ^)^. In addition, access to remotely located stores in the FSU may be limited due to the lack of convenient and affordable public transport and scarcity of cars. Nevertheless, one can be more optimistic about a causative interpretation of our findings because regional fixed effects are also included in the analysis, which should account for interregional variations in socio-economic indicators. In another middle-income country – Brazil – a study that also controlled for area socio-economic status found a similar positive correlation between regular fruit and vegetable intake and density of food markets specializing in fruit and vegetables^(^ ^40^ ^)^.

It should also be mentioned that the consequences of better access to supermarkets can differ from those of better access to convenience stores, and that our data set does not make a clear distinction between these two kinds of stores. Thus, some studies have found either no or negative associations between availability of convenience stores and fruit and vegetable consumption^(^ ^38^ ^,^ ^39^ ^)^. This can be because such stores may provide less choice of fresh fruit and vegetables, and thus encourage people to buy more unhealthy food items. Alternatively, such stores may be located in more economically disadvantaged areas and thus the observed association between dietary patterns and convenience store access may be partly driven by variations in neighbourhood socio-economic status. Although there is no proxy for convenience store availability, there is a variable measuring the number of stores selling sweets and crisps in the neighbourhood. While there is no significant association between this variable and daily fruit or vegetable consumption for the whole sample and women only, surprisingly it is significant and positive for men. Nevertheless, it is important to emphasize that these stores may not necessarily be limited in their supply of fruit or vegetables (and thus not properly fall in the category of convenience stores) and therefore one should not over-interpret this finding.

### Nutrition and advertising

The relationship between food advertising and dietary behaviours is also of interest, as sums spent on advertising are very large, most promoting unhealthy foods^(^ ^41^ ^)^. However, the existing literature on the effect of food advertising on either fruit or vegetable consumption is limited and tends to focus on adolescents, as well as on television advertising only^(^ ^42^ ^)^. A considerable part of this literature is based on small-scale experimental studies of questionable generalizability.

We find billboard advertising of snacks and sweet drinks (including juices) to be significantly negatively related to daily fruit or vegetable consumption. While this does not prove that billboard advertising for unhealthy foods causes less consumption of fruit or vegetables (as it may well be that such advertisements are deliberately placed in communities where unhealthy eating is more prevalent), the fact that the effect is significant even with the inclusion of regional effects, as well as of a range of both community and individual controls, does increase confidence in our findings. Also the fact that this association is much stronger for women in both cases suggests that local confounders are unlikely to be the main explanation. Surprisingly, billboard adverts for soft drinks are positively related to daily fruit or vegetable consumption (although not for men). One can speculate that the positive sign found for soft drinks advertising might be due to a complementarity or substitutability between fruit and vegetables, and other goods. For instance, while juice drinks could be perceived as substitutes for fruit and so consumed as an alternative, soft drinks or sweets could instead be more often consumed with fruit. We could not find any other studies that measured this association.

### Fast food and restaurants

The role played by availability and access to fast-food outlets is also unclear. Thus, although, theoretically, one can expect easier access to fast-food stores to be associated with worse dietary patterns^(^ ^43^ ^)^, such findings may be due to community-level confounding by neighbourhood socio-economic status, with less well-off communities more likely to provide access to fast-food establishments.

Empirical evidence on this topic has so far been inconclusive. One New Zealand study found neighbourhood access to fast-food establishments unrelated to fruit and vegetable consumption^(^ ^38^ ^)^. The previously cited study from Brazil found no association between fast-food outlet density and fruit and vegetable intake^(^ ^40^ ^)^. Several US studies found easier access to fast-food outlets to be negatively related to diet-related outcomes^(^ ^38^ ^)^. Our finding of a significant negative association between the ease of access to fast-food outlets and the probability of daily fruit or vegetable consumption for men is thus more consistent with the US studies.

As for easier access to full-service restaurants, theoretically it is unclear how they may affect dietary attitudes and behaviours. On one hand, the effect may depend on the food choice on offer (traditional menus are quite heavily meat- and potato-based in many FSU countries). Conversely, any empirical finding of an association between these variables should be tempered by the risk of confounding by neighbourhood-level characteristics. As it is, the existing empirical evidence is more limited than for fast-food outlets. One study found, for example, that better access to a full-service restaurant was related to lower intake of saturated fat among black Americans^(^ ^41^ ^)^. Another study reached a different conclusion after finding that away-from-home eating (with both restaurant and fast-food consumption) was related to worse quality of diet^(^ ^22^ ^)^. Our finding of a small positive association between the number of local food restaurants and greater fruit or vegetable consumption in the FSU countries (especially for women) adds to this growing literature.

### Data limitations

Although our rich data set helps alleviate potential endogeneity concerns, there are certain limitations. The questionnaires were not primarily designed to assess diet and only recorded whether respondents had eaten any fruit or vegetables during the past week and not how often. Although eating fruit or vegetables daily or almost daily may still not guarantee that adult people eat their recommended amount of 400 g/d, at least this group is more likely to meet fruit and vegetable targets. The need for data collected with food frequency or dietary recall questions is clear^(^ ^44^ ^)^.

In addition, the fruit and vegetable consumption variables have not been validated for the HITT study. Having said that, very similar variables have been used in another published article^(^ ^45^ ^)^. A possible concern regarding the external validity of our results comes from the fact that data were collected in the spring, between March and May, a period when fruit and vegetables will be in relatively poor supply compared with June to September. This timing may lead us to underestimate the effect of proximity to stores on fruit and vegetable consumption.

Also, observed associations may not be causal. For example, community-level exposure to advertising may be determined by the perceived attractiveness of the neighbourhood demographics to marketing organizations and placement of stores may also depend on the perceived wealth of the community. Having said that, this issue is addressed in two main ways: (i) as all the variables of interest are included in equation (1) simultaneously, partial regression coefficients obtained for each covariate demonstrate the association adjusted for any potential confounding by observable variables; and (ii) by including community fixed effects in equation (1), and regional fixed effects in equation (2), any additional area-level confounding affecting both the covariates of interest and the outcome variable is controlled for.

Some of the community-level indicators may also be imperfect measures of the variables of interest. Thus, the data set lacks information on size of outlets. Moreover, it is possible that the same outlet may sell both healthy (e.g. fruit and vegetables) and unhealthy (e.g. biscuits) items. Nevertheless, given these limitations, it is encouraging that our results are largely consistent with prior expectations.

## Conclusions

The present study is the first one to examine both the individual- and community-level determinants of fruit and vegetable consumption in nine FSU countries. It confirms the inadequacy of consumption in this region and sheds light on which groups are most vulnerable: namely men, those at older ages, with less education and fewer financial resources. However, beyond these individual attributes, the local food economy also plays a role. Taken together, these findings provide potential entry points for policy interventions.

## Supplementary material

For supplementary material accompanying this paper visit http://dx.doi.org/10.1017/S1368980015000105. click here to view supplementary material
