# Supplementary material for: Fruit and vegetable consumption in the former Soviet Union: the role of individual- and community-level factors
Source: Public Health Nutr. 2015 Feb 17;18(15):2825–35. doi: 10.1017/S1368980015000105 (PMC4595861; doi:10.1017/S1368980015000105)
Supplement: Supplementary file 1 [file S1368980015000105sup001.doc]

**Supplementary material**

**Annex 1.** Variable definitions

| **Variable name** | **Original questions** | **Variable definitions** |
| --- | --- | --- |
| Fruit consumption daily/almost daily (excluding juice) | How often in the past week have you eaten the following? | Select daily/almost daily from the following 4 categories:  -Daily/almost daily  -Several times a week  -Once a week  -Less than once a week. |
| Vegetable consumption daily/almost daily (excluding potatoes) | How often in the past week have you eaten the following? | Select daily/almost daily from the following 4 categories:  -Daily/almost daily  -Several times a week  -Once a week  -Less than once a week. |
| Primary education | What is your highest level of education? | Select "Primary or without education" and "Incomplete secondary basic" from the following:  -Primary or without education  -Incomplete secondary basic  -Completed secondary education including vocational  -Completed secondary special college  - Non-finished higher education  - Completed higher education |
| Secondary education | What is your highest level of education? | From the above list for primary education, select:  -Completed secondary education including vocational  -Completed secondary special college |
| Tertiary education | What is your highest level of education? | From the above list for primary education, select:  - Non-finished higher education; Completed higher education |
| Good economic situation? | How would you describe the economic situation of your household at present time? | Select from 5 options:  -Very good and Good |
| Household size | Including you, how many people constantly live in this household including children and adults? | n/a |
| Married | What is your marital status? | Select "married/co-habiting" from the following list:  -never married  -married or co-habiting  -divorced  -widow/er in the last 5 years  -widow/er for more than 5 years |
| Village | Type of living location. | Select village from :  -village; capital city; regional capital; city; urban settlement |
| Capital | Type of living location. | Select capital city from:  -village; capital city; regional capital; city; urban settlement |
| Assets | See Annex 2 section for extended definition | n/a |
| Good diet not important | Please indicate your agreement with the statements about ways of maintaining good health. | Select from 4 options: healthy diet is unimportant |
| Number of adverts separately for fast food, snacks, sweet drinks/juices; soft drinks. | Count the number of advertisement you see and mark down in each category. Advertisements include that on billboards, pasted on shop windows, bus shelters or other locations that is easily visible from the street. | n/a |
| Number of shops selling crisps and sweets | Count the number of shops / outlets you see that sell the following and mark down in each category. | n/a |
| Number of shops selling fruit and vegetables | Count the number of shops / outlets you see that sell the following and mark down in each category. | n/a |
| Number of local food restaurants | Count the number of outlets you see that sell the following and mark down in each category. | n/a |
| Easy walk to fast food outlet? | Can people living in this community access fast food outlets? | Select: Yes, within an easy walk from the following options:  easy walk;  =<30 minutes by transport; =>30 minutes by transport |
| -garbage is collected by authorities from all homes?  -cold water supply?  -central steaming systems? | What proportion approximately of homes in this community have the following facilities see on the left? | Select: None from:  -none; less than 50%; more than 50% but not all; all |

**Annex 2.** Asset classes definition

We created dummy variables for being in one of 4 asset classes as proxies for household wealth, following the approach suggested in ^44^, with 25%, 50% and 75% cut-offs. Specifically, for each of the selected assets *i* fridge, TV, mobile phone, computer, old car, new washing machine, new car in each country *c*, we created country and asset-specific weights *w_i_*, equal to the reciprocal of the population owning the corresponding assets in each country. For each respondent *p* living in country *c*, we then multiplied the country and asset-specific weight *w_i_*, by indicators for whether they had the asset *A_i_*, and summed this over all assets:

*Score_pc_* =$\sum_{i} w_{i}\times A_{i}$ 3

Finally, we created dummy variables for being in 4 separate asset classes, depending on 25%, 50% and 75% cut-offs of the asset index score.

**Annex 3. Proportion of respondents who agreed with the statement that good diet is not important for health**

| Country | Proportion, % |
| --- | --- |
|  |  |
| Armenia | 0.67% |
| Azerbaijan | 0.56% |
| Belarus | 0.95% |
| Georgia | 0.51% |
| Kazakhstan | 1.51% |
| Kyrgyzstan | 1.11% |
| Moldova | 0.11% |
| Russia | 1.85% |
| Ukraine | 0.86% |
